# Effects of immunosuppression on tracheal mucosal responses after infection with *Mycoplasma gallisepticum* in vaccinated and unvaccinated chickens

**DOI:** 10.3389/fimmu.2026.1802515

**Published:** 2026-07-23

**Authors:** Sathya N. Kulappu Arachchige, Anna Kanci Condello, Amir H. Noormohammadi, Kelly A. Tivendale, Glenn F. Browning, Nadeeka K. Wawegama

**Affiliations:** 1 Asia-Pacific Centre for Animal Health, Melbourne Veterinary School, Faculty of Science, The University of Melbourne, Parkville, VIC, Australia; 2 Asia-Pacific Centre for Animal Health, Melbourne Veterinary School, Faculty of Science, The University of Melbourne, Werribee, VIC, Australia

**Keywords:** *Mycoplasma gallisepticum*, RNA-sequencing, immunosuppression, chicken anaemia virus, CAV, infectious burial disease virus, IBDV, trachea

## Abstract

**Introduction:**

Vaccination is the most common method used to control infection caused by *Mycoplasma gallisepticum* in chickens. However, concurrent immunosuppression may compromise vaccine efficacy.

**Methods:**

This study investigated the effect of immunosuppression induced by either chicken anaemia virus (CAV) or infectious bursal disease virus (IBDV), administered prior to or following vaccination with the *M. gallisepticum* live vaccine strain ts-304 (Vaxsafe MG304), on tracheal host responses to subsequent challenge with virulent *M. gallisepticum*. Tracheal responses were assessed by genome-wide transcriptional profiling in these groups and compared across unvaccinated-unchallenged, unvaccinated-challenged, vaccinated-unchallenged and vaccinated-challenged groups that had not been exposed to CAV or IBDV.

**Results:**

Immunosuppression resulted in significant differences in tracheal transcriptional responses compared to immunocompetent groups, irrespective of the timing of infection with CAV or IBDV. Differences in transcription were more pronounced in the IBDV-infected groups than the CAV-infected groups. Functional interrogation of differentially transcribed genes revealed adverse effects of *M. gallisepticum* on extracellular and intracellular signalling, cell communication, cellular actin dynamics, formation of the cellular cytoskeleton, and cellular metabolic pathways in the tracheal mucosa of immunosuppressed groups. In addition, there were indications of down-regulation of both innate and adaptive immune responses, including cytokine signalling, antigen processing presentation, and immune cell receptor signalling in the immunosuppressed groups. Specifically, findings indicated that infection with CAV impaired pro-inflammatory and adaptive T-cell responses in the tracheal mucosa.

**Conclusions:**

The findings implied that the primary immune response induced post-vaccination and the secondary immune response induced post-challenge with virulent *M. gallisepticum* were both affected by infection with these two viruses. Coupled with previous observations of reduced antibody titres against *M. gallisepticum*, and increased rates of recovery of virulent *M. gallisepticum* in both CAV- and IBDV-infected groups, the significant transcriptional changes detected in the current study highlighted the roles of both cell-mediated (CMI) and humoral immunity (HI) in vaccine-induced protection, as CAV mainly affects CMI and IBDV mainly affects HI. These findings will assist in identifying the mechanisms underlying the reduced efficacy of live attenuated mycoplasma vaccines in immunosuppressed animals.

## Introduction

*Mycoplasma gallisepticum* is a globally significant bacterial pathogen of poultry, causing chronic respiratory disease in chickens, infectious sinusitis in turkeys (1) and conjunctivitis in house finches (2). In chickens, the disease is characterised by inflammation of the upper and lower respiratory tract (3, 4), which results in clinical signs of coughing, sneezing, râles, and nasal and ocular discharge (5, 6). The consequent reduction in productivity causes significant economic loss in breeder, layer and broiler chicken flocks due to reduced feed consumption, lower weight gain, reduced egg production and hatchability, and increased carcass condemnation (5–7).

Chicken anaemia virus (CAV) and infectious bursal disease virus (IBDV) cause immunosuppression in chickens, predisposing them to infections with other agents (8, 9). Previous studies examining the effect of immunosuppression on the pathogenesis of *M. gallisepticum* have found that it results in more severe pathology and increased bacterial loads in the respiratory tract (10, 11). Moreover, in a previous study we found that immunosuppression caused by infection with CAV or IBDV reduced the efficacy of vaccination with the live attenuated *M. gallisepticum* vaccine strain ts-304 (12).

Host responses in the trachea after vaccination and/or infection with *M. gallisepticum* (13–16), and after coinfection with *M. gallisepticum* and low-pathogenicity avian influenza A virus (17), have been investigated previously using genome-wide transcriptome analysis, with the aim of understanding the transcriptional changes associated with maladaptive and protective host responses in these infection/vaccination models. Similarly, the gene transcriptional profiles in the lymphoid organs, including the thymus, bone marrow, spleen and bursa of Fabricius after infection with CAV (18, 19), and the bursa of Fabricius and spleen after infection with IBDV (20–25), have been explored using RNA-seq or microarrays. However, the effect of immunosuppression caused by infection with CAV or IBDV on genome-wide transcriptional changes in the target organs of other pathogens, including *M. gallisepticum*, have not been investigated previously.

In the study described here, the global gene transcriptional profiles were examined in the tracheal mucosal tissues of chickens that had been vaccinated against *M. gallisepticum* and subsequently challenged with wild type *M. gallisepticum* after immunosuppression by infection with either CAV or IBDV at the time of vaccination or at the time of challenge. These transcriptional profiles were compared to those of unvaccinated chickens that had been challenged with the wild type strain (challenged-only), to unvaccinated chickens that remained unchallenged (negative-control), chickens that had been vaccinated against *M. gallisepticum* and challenged with the strain Ap3AS (vaccinated-and-challenged), and chickens that had been vaccinated but remained unchallenged (vaccinated-only), with the aim of understanding the effect of immunosuppression on transcriptional changes after challenge with virulent *M. gallisepticum* in the tracheas of chickens vaccinated against *M. gallisepticum*.

## Methods

### Experimental vaccination and infection of chickens

The procedures used for experimental infection with CAV and IBDV, vaccination with the attenuated *M. gallisepticum* strain ts-304 and challenge with virulent *M. gallisepticum* have been described previously (12). Briefly, 80 White Leghorn specific-pathogen-free (SPF) chickens were randomly allocated into eight different groups of 10: Group 1, negative-control; Group 2, vaccinated-only; Group 3, challenged-only; Group 4, vaccinated-and-challenged; Group 5, CAV-vaccinated-challenged; Group 6, vaccinated-CAV-challenged; Group 7, IBDV-vaccinated-challenged; and Group 8, vaccinated-IBDV-challenged (**Supplementary Figure 1**). Each group was kept in a separate, microbiologically secure isolator with feed and water provided *ad libitum*. At 1 week of age, birds in Group 5 were inoculated by eye drop with 10^4^ median tissue culture infectious doses (TCID_50_) of CAV strain CAU269/7 and birds in Group 7 were inoculated by eye drop with 10^3^ TCID_50_ of IBDV strain 002/73. At 3 weeks of age, the birds in Group 2 and Groups 4-8 (all groups except the negative-control and challenged-only groups) were vaccinated by eye drop with 30 µl of reconstituted, freeze-dried vaccine (Vaxsafe**^®^** MG304) containing 10^6.0^ colour changing units (CCU) of *M. gallisepticum* ts-304. At 6 weeks of age, birds in Groups 6 and 8 were inoculated by eye drop with CAV or IBDV, respectively, using the same doses and strains that were used to inoculate Groups 5 and 7. Five weeks after vaccination, birds from Groups 3-8 (all groups except the negative-control and vaccinated-only groups) were exposed to aerosols of a *M. gallisepticum* culture containing 10^6.5^ CCU/ml of the wild-type strain Ap3AS using an established protocol (26). Two weeks after challenge, all birds were euthanised via intravenous injection of pentobarbitone (160 mg/kg) and necropsied. All procedures involving animals were reviewed and approved by the University of Melbourne Animal Ethics Committee under approval number 1914907.1.

Throughout this paper, “vaccination” indicates inoculation by eye drop with *M. gallisepticum* strain ts-304, “challenge” indicates experimental infection with *M. gallisepticum* wild-type strain Ap3AS by aerosol exposure, and “infection” indicates experimental infection with either CAV or IBDV by eye drop inoculation. Further, the four groups infected with either CAV or IBDV were collectively classified as “immunosuppressed”, whereas the groups not infected with either virus were classified as “immunocompetent”.

### Total RNA extraction and sequencing

Tracheal cross sections approximately 1–2 cm in length were collected from each bird into RNAlater stabilisation solution (Invitrogen, Carlsbad, CA, USA) and stored at -20 °C. Total RNA was extracted from the stabilised tracheal mucosal tissues of three randomly chosen chickens from each of the negative-control, vaccinated-only, challenged-only and vaccinated-and-challenged groups, and all ten birds in the four immunosuppressed groups (one sample from the vaccinated-IBDV-challenged group was not included in the study because of RNA degradation), as described previously (15). Total RNA extracted from the tracheal mucosal tissues from all birds in the immunosuppressed groups were included in this study, instead of three randomly chosen birds, as greater variability in the tracheal lesions was detected within each immunosuppressed group in previous histopathological studies (12). Briefly, the total RNA was extracted from each tracheal mucosal tissue homogenate using the RNeasy Mini Kit (QIAGEN, Hilden, Germany) and then treated with DNase using the TURBO DNA-*free* kit (Invitrogen), prior to cleaning and concentrating using the Zymo RNA Clean & Concentrator-25 kit (Zymo Research Corporation, Irvine, CA, USA) as recommended by the manufacturers. The quality of the RNA was confirmed using an Agilent 4200 TapeStation system (Agilent Technologies, Santa Clara, CA, USA) and samples with RNA integrity numbers (RIN) > 8 were further processed for sequencing. The cDNA libraries were then constructed from 300–500 ng of total RNA from each tracheal sample using a TruSeq Stranded mRNA Library Preparation kit (Illumina Inc., San Diego, CA, USA) according to the manufacturer’s instructions. The quality of each of the 52 uniquely indexed libraries was confirmed using the Agilent 2200 TapeStation system and these libraries were then normalised to 1 nM before pooling them. The pooled libraries were sequenced on a NextSeq500 sequencing platform (Illumina) to obtain 150 bp paired-end reads.

### Pre-processing of RNA-seq reads

The quality of RNA-seq reads derived from individual libraries was assessed using FastQC (version 0.11.9, https://www.bioinformatics.babraham.ac.uk/projects/fastqc/) and the Illumina sequencing adapter sequences and bases with a PHRED score of ≤ 20 were then removed using Trim Galore (version 0.6.4, https://www.bioinformatics.babraham.ac.uk/projects/trim_galore/). Further quality control was performed by removing non-paired reads using BBMap (version 38.86, https://sourceforge.net/projects/bbmap/). The gff3, gtf and fasta files of the annotated *Gallus gallus* (chicken) genome were downloaded from release 101 of the Ensembl database (27) and the mRNA sequences for protein-coding genes were extracted. The mRNA sequences were then indexed and used for high-quality paired-end RNA-seq mapping (28) using bowtie2 version 2.3.5 (29), and the expected read count per gene was calculated using RSEM version 1.3.3 (28).

### Differential gene transcription analysis

The analysis of RNA-seq data representing different groups of chickens was conducted using limma version 3.40.6 (30), Glimma version 1.12.0 (31) and edgeR version 3.26.8 (32) in the RStudio environment (version 1.2.5019, http://www.rstudio.com/) following recommended guidelines (33). For each biological replicate, the read counts for each gene, as enumerated using RSEM, were used as input data for edgeR. The levels of differential transcription were calculated based on pairwise comparisons between groups (that is, each group versus every other group). Briefly, read counts were first converted into counts-per-million (CPM) to standardise for differences in library size and genes with low levels of transcription, at a threshold CPM value of ≤ 1 in ≥ 3 samples, were excluded from further analyses. The trimmed mean of M-values (TMM) method was then used to normalise the data for differences in distributions of transcripts between samples (34). Heteroscedascity was removed from count data using voom (35) and CPMs were log_2_ transformed. A linear model was fitted to the normally distributed log_2_ CPM values after accommodating the mean-variance relationship using precision weights calculated using the voom function (35) to compare gene transcription between the groups. Genes that had a log_2_ (1) fold (i.e., 2-fold) change in transcription at a false discovery rate (FDR) of < 0.05 were considered significantly differentially transcribed. Differentially transcribed genes are reported as up- or down-regulated in the first group compared to the second group in each pair-wise comparison.

### Functional annotation and integration

Functional gene ontologies (GO) enriched with up- or down-regulated genes in each pair-wise comparison were identified using topGO version 2.36.0 (36), as described previously (16). Briefly, a *G. gallus* GO universe was constructed using GO-IDs linked to each protein-coding gene of *G. gallus*, extracted using the biomaRt annotation tool version 2.40.0 (37) to identify molecular functions (MFs), biological processes (BPs) and cellular components (CCs) enriched with up- or down-regulated genes. GO terms significantly enriched with up- or down-regulated genes were further summarised by removing redundant GO terms using the REVIGO web server (38). Identification of biological pathways and protein classes enriched with up- or down-regulated genes was performed using the Reactome and Panther databases available in the PANTHER classification tool version 14.1 (39). Gene ontologies enriched at a FDR of < 0.01, and pathways and protein classes enriched at a FDR of < 0.05 were regarded as significant. The GO terms, Reactome pathways and Panther protein class analyses were conducted only for the pair-wise comparisons for which significant differences in transcription were detected.

## Results

### The challenged-only group and the immunosuppressed groups had significant differences in transcription compared to other groups

RNA-seq reads were mapped to 16,779 *G. gallus* protein-coding genes, and 13,576 genes with a count per million (CPM) of > 1 in three or more samples were retained for differential gene transcription comparisons between the groups.

**Figure 1:**
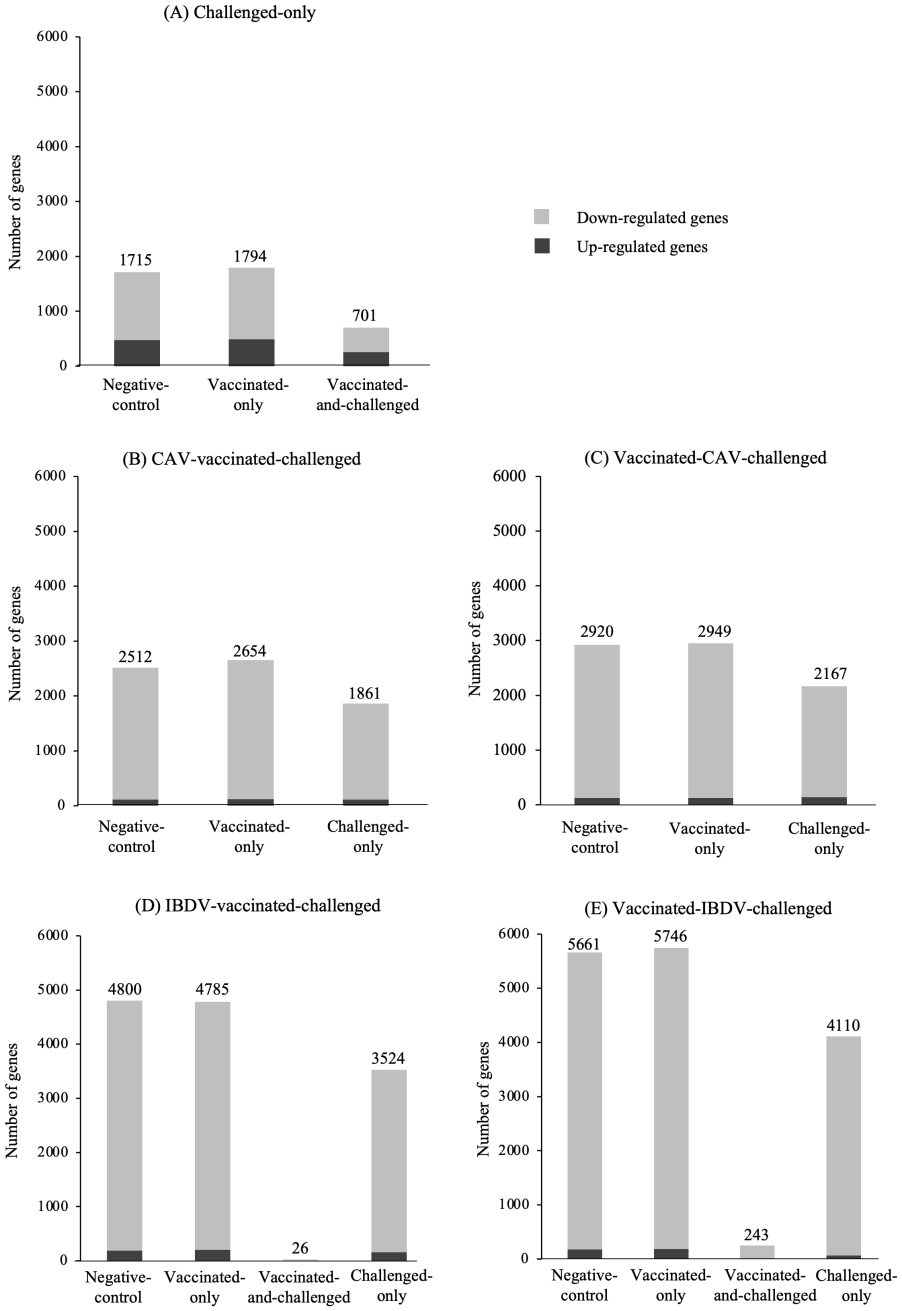
Differentially transcribed genes detected in pair-wise comparisons between the groups included in this study. **(A)** challenged-only group, **(B)** CAV-vaccinated-challenged group, **(C)** vaccinated-CAV-challenged group, **(D)** IBDV-vaccinated-challenged group and **(E)** vaccinated-IBDV-challenged group compared with the groups shown along the x-axis of each graph. No significantly differentially transcribed genes were identified in either of the CAV-infected groups when they were compared with the vaccinated-and-challenged group, so these comparisons are not shown in panels **(B)** and **(C)**.

At a FDR of < 0.05, differentially transcribed genes were detected in the challenged-only group in comparison with the negative-control (1,715 genes), vaccinated-only (1,794 genes) and vaccinated-and-challenged (701 genes) groups (**Figure 1A**). A total of 2,512 differentially transcribed genes were detected in the CAV-vaccinated-challenged group in comparison with the negative-control group, 2,654 genes compared to the vaccinated-only group and 1,861 genes compared with the challenged-only group (**Figure 1B**). In the vaccinated-CAV-challenged group, differentially transcribed genes were detected compared to the negative-control (2,920 genes), vaccinated-only (2,949 genes), and the challenged-only (2,167) groups (**Figure 1C**). Similarly, a total of 4,800 genes were differentially transcribed in the IBDV-vaccinated-challenged group in comparison with the negative-control group, 4,785 genes compared to the vaccinated-only group and 3,524 genes compared with the challenged-only group. In addition, 26 genes had significant differences in transcription in the IBDV-vaccinated-challenged group compared with the vaccinated-and-challenged group (**Figure 1D**). In the vaccinated-IBDV-challenged group, a total of 5,661 genes were differentially transcribed in comparison with the negative-control group, 5,746 genes compared to the vaccinated-only group, 243 genes compared with the vaccinated-and-challenged group and 4,110 genes compared with the challenged-only group (**Figure 1E**).

Out of the four immunosuppressed groups, more genes were differentially transcribed in the two IBDV-infected groups (**Figures 1D, E**) in comparison with other groups than in the two CAV-infected groups (**Figures 1B, C**) in comparison with other groups. In all pair-wise comparisons, more genes were down-regulated than being up-regulated. The numbers of up- and down-regulated genes in each pairwise comparison are listed separately in **Supplementary Table 1**.

### There were three distinct clusters of groups based on their transcriptional profiles

**Figure 2:**
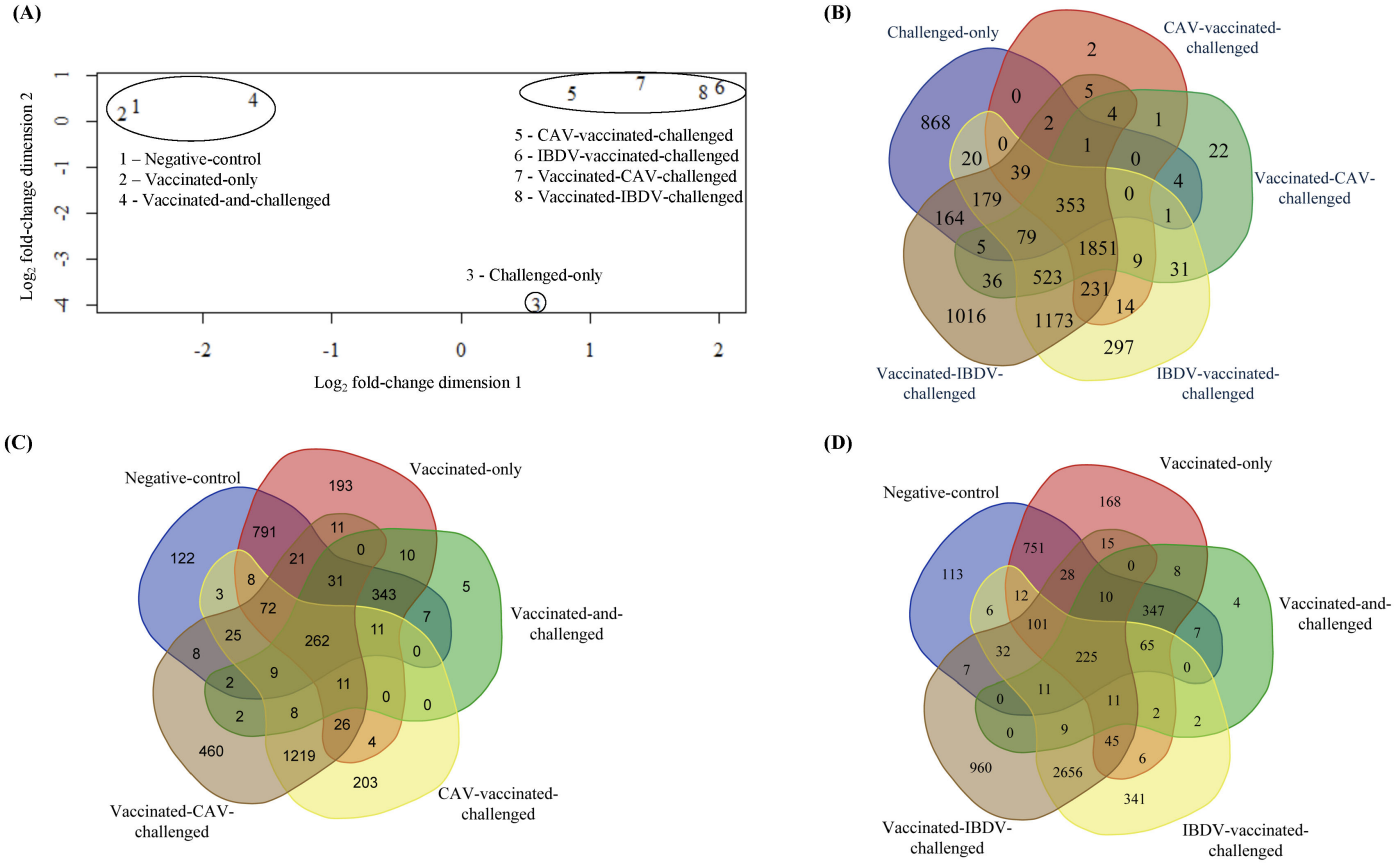
**(A)** Two-dimensional MDS plot based on the log_2_ counts-per-million. Distances on the plot correspond to the average log_2_ fold-change for each gene between each pair of groups. **(B)** The number of differentially transcribed genes in the challenged-only, CAV-vaccinated-challenged, vaccinated-CAV-challenged, IBDV-vaccinated-challenged and vaccinated-IBDV-challenged groups in comparison with the negative-control group. **(C)** The number of differentially transcribed genes in the challenged-only group in comparison with the negative-control, vaccinated-only, vaccinated-and-challenged, CAV-vaccinated-challenged and vaccinated-CAV-challenged groups. **(D)** The number of differentially transcribed genes in the challenged-only group in comparison with the negative-control, vaccinated-only, vaccinated-and-challenged, IBDV-vaccinated-challenged and vaccinated-IBDV-challenged groups.

Unsupervised relationship plots generated based on the mean log_2_ CPM values of each group for each gene revealed three distinct clusters: one cluster comprising the negative-control, vaccinated-only and vaccinated-and-challenged groups; a second cluster comprising the four immunosuppressed groups; and a third cluster comprising only the challenged-only group (**Figure 2A**). The same finding was evident after visualisation of the number of differentially transcribed genes that were unique or common to different pair-wise comparisons. In the challenged-only group 868 genes were uniquely differentially transcribed in comparisons with the negative-control group, while 1851 differentially transcribed genes were common to the comparisons of each of the immunosuppressed groups with the negative-control group (**Figure 2B**). A total of 1219 genes were differentially transcribed in the challenged-only group in comparisons with each of the CAV-infected groups, while a different set of genes were differentially transcribed in the challenged-only group in comparisons with the negative-control, vaccinated-only, and vaccinated-and-challenged groups (**Figure 2C**). A similar trend was observed in comparisons of the challenged-only group with each of the IBDV-infected groups, and the negative-control, vaccinated-only and vaccinated-and-challenged groups (**Figure 2D**).

### Gene ontologies related to RNA and protein metabolism, and cellular respiration, were significantly enriched with genes up-regulated in comparisons of the immunosuppressed groups with the negative-control and vaccinated-only groups

Functional GO analysis identified GOs significantly enriched with genes up- or down-regulated in each pair-wise comparison. The numbers of cellular components, molecular functions, and biological processes enriched with up- or down-regulated genes in each immunosuppressed group compared to the negative-control group, and the vaccinated-only group, are shown in **Table 1**.

**Table 1:** Number of cellular components (CCs), molecular functions (MFs) and biological processes (BPs) significantly enriched with up- or down-regulated genes in the first group compared with the second group in each pair-wise comparison.

Comparison	Up			Down		
	CC	MF	BP	CC	MF	BP
CAV-vaccinated-challenged vs negative-control	43	7	50	133	94	563
CAV-vaccinated-challenged vs vaccinated-only	51	15	70	142	99	677
Vaccinated-CAV-challenged vs negative-control	51	13	73	115	89	463
Vaccinated-CAV-challenged vs vaccinated-only	52	13	81	125	103	657
IBDV-vaccinated-challenged vs negative-control	55	26	90	141	111	691
IBDV-vaccinated-challenged vs vaccinated-only	57	26	95	150	123	861
Vaccinated-IBDV-challenged vs negative-control	56	25	91	162	133	792
Vaccinated-IBDV-challenged vs vaccinated-only	59	18	87	158	138	802
CAV-vaccinated-challenged vs challenged-only	2	2	1	110	96	736
Vaccinated-CAV-challenged vs challenged-only	11	0	10	114	87	647
IBDV-vaccinated-challenged vs challenged-only	1	1	2	138	124	912
Vaccinated-IBDV-challenged vs challenged-only	2	2	5	126	137	798
Challenged-only vs negative-control	32	14	260	44	13	90
Challenged-only vs vaccinated-only	26	27	272	46	11	110
Challenged-only vs vaccinated-and-challenged	21	17	182	29	7	52

The REVIGO non-redundant functional GO summaries of the up-regulated genes in each of the four immunosuppressed groups compared with the negative-control and vaccinated-only groups had similar profiles in the cellular component, molecular function, and biological process GO categories. Briefly, cellular components enriched with up-regulated genes included ribonucleoprotein complex, protein-containing complex, cytosol, cytoplasm, non-membrane bounded organelle, respirasome and envelope (**Supplementary Figures 2**-**5**). The molecular functions enriched with up-regulated genes were rRNA-binding, structural constituent of ribosome, structural molecular activity, oxidoreductase activity-acting on a haem group of donors, and proton transmembrane transporter activity (**Supplementary Figures 6**-**9**). The biological processes enriched with up-regulated genes included ribosome biogenesis, metabolic processes, cellular-amide metabolic process, positive regulation of signal transduction by p53 class mediator, and proton transmembrane transport (**Supplementary Figures 10**-**13**).

### Gene ontologies related to organisation of chromatin, cell junction and cellular cytoskeleton, cell migration, cell cycle and cellular signalling were significantly enriched with genes down-regulated in the immunosuppressed groups compared with the negative-control and vaccinated-only groups

The REVIGO non-redundant functional GO summaries of the genes down-regulated in the four immunosuppressed groups compared with the negative-control and vaccinated-only groups had similar profiles in the cellular component, molecular function, and biological process GO categories. Briefly, cellular components enriched with down-regulated genes included those related to anchoring junctions, membrane trafficking, extracellular matrix, chromosome, cytoskeleton and DNA-repair complex (**Supplementary Figures 14**-**17**). The molecular functions enriched with down-regulated genes were those related to molecular binding, phosphotransferase activity, cytoskeletal motor activity, semaphorin receptor activity, transcription co-regulatory activity, guanyl-nucleotide exchange factor activity, and extracellular matrix structural constituent (**Supplementary Figures 18**-**21**). The biological processes enriched with down-regulated genes included chromosome organisation, cell junction organisation, cellular cytoskeleton organisation, cell migration, cell cycle, regulation of GTPase activity, cellular signalling and communication (Wnt/JNK, MAPK, ERBB, NF-κB and apoptotic signalling), and protein modification and transport (**Supplementary Figures 22**-**25**). The biological process circulatory system development was enriched with down-regulated genes only in the two CAV-infected groups, and cilium and cell junction organisation were enriched with down-regulated genes only in the IBDV-infected groups, in comparison with the negative-control and vaccinated-only groups.

### Gene ontologies related to cilium and cytoskeleton organisation, and receptor binding, were enriched with genes up-regulated genes in the immunosuppressed groups compared with the challenged-only group

The numbers of cellular components, molecular functions, and biological processes enriched with up- or down-regulated genes in each immunosuppressed group compared to the challenged-only group are shown in **Table 1**. The cellular components enriched with up-regulated genes included those related to cilium, cytoskeleton and cellular respiratory chain (**Supplementary Figures 26**-**29**). The molecular functions enriched with up-regulated genes were those related to chemokine receptor binding, protein binding and peroxidase activity (**Supplementary Figures 30**-**32**). The biological processes enriched with up-regulated genes included cilium organisation/assembly, microtubule-based process, antibiotic catabolic process, defence response to bacterium and hydrogen peroxide metabolic process (**Supplementary Figure 33**-**36**).

### Gene ontologies related to DNA replication, cell proliferation and migration, and immune response were enriched with genes down-regulated in the four immunosuppressed groups compared with the challenged-only group

The REVIGO non-redundant functional GO summaries of the down-regulated genes in the four immunosuppressed groups compared with the challenged-only group had similar profiles in the cellular component, molecular function, and biological process GO categories. Briefly, cellular components enriched with down-regulated genes included ribonucleoprotein complex, nuclear chromosome, spindle, lamellipodium, vesicle, and endoplasmic reticulum (**Supplementary Figures 37**-**40**). The molecular functions enriched with down-regulated genes were those for protein, nucleic acid, histone, chromatin, ion, carbohydrate-derivative binding, and ribonucleoprotein complex binding, helicase activity, and C-C chemokine receptor activity (**Supplementary Figures 41**-**44**). The biological processes enriched with down-regulated genes included DNA metabolism, regulation of immune response, lymphocyte activation, leukocyte migration, defence response, regulation of leukocyte proliferation, leukocyte cell-to-cell adhesion, mitosis, cell death, apoptosis, and regulation of hydrolase activity (**Supplementary Figures 45**-**48**).

### Reactome pathways involved in RNA and protein metabolism, citric acid cycle, mitochondrial biogenesis, and DNA repair were enriched with genes up-regulated in the immunosuppressed groups compared with the negative-control and vaccinated-only groups

**Figure 3:**
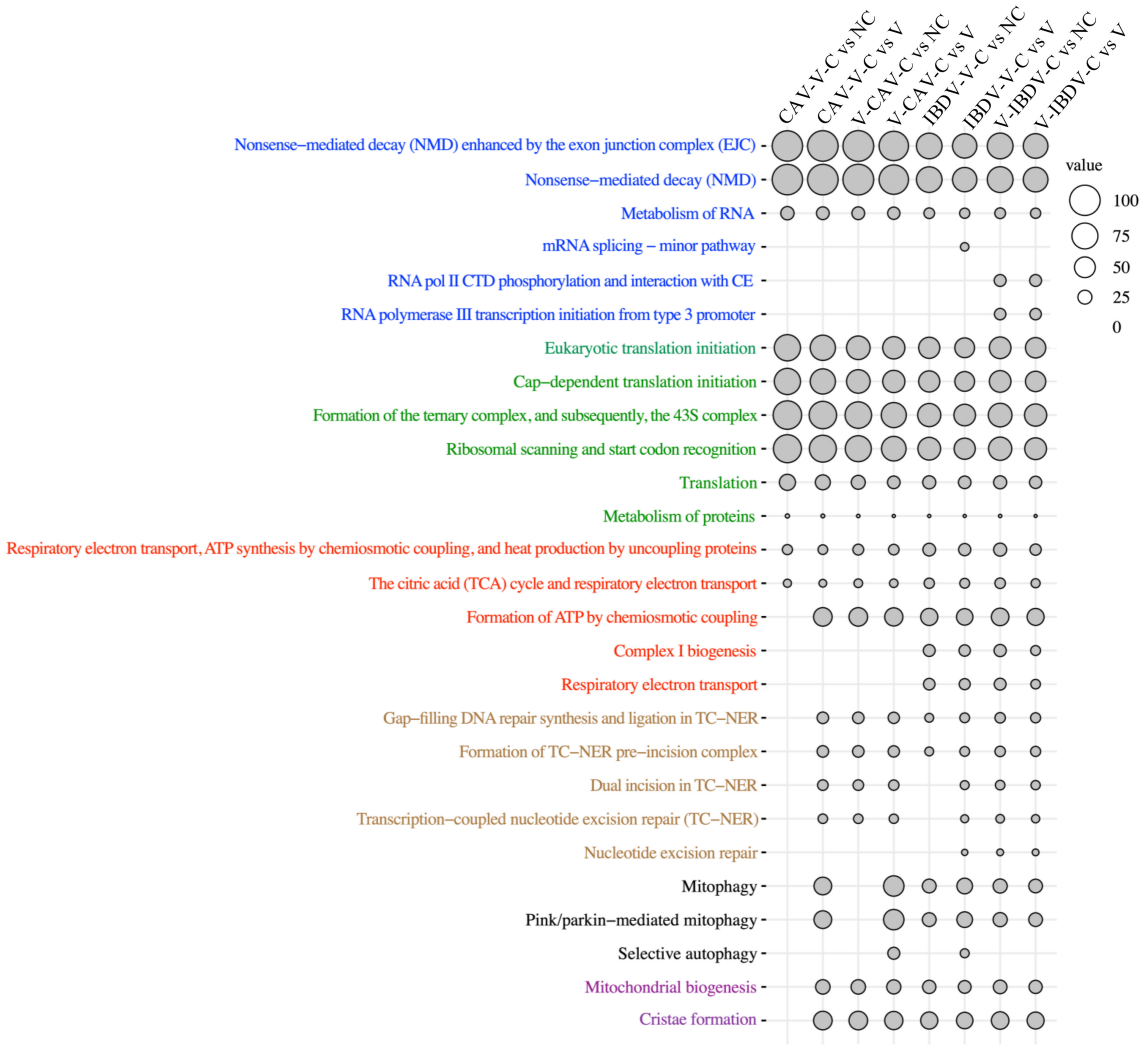
Reactome pathways enriched with up-regulated genes in the four immunosuppressed groups (CAV-V-C; CAV-vaccinated-challenged, V-CAV-C; vaccinated-CAV-challenged, IBDV-V-C; IBDV-vaccinated-challenged, V-IBDV-C; vaccinated-IBDV-challenged) compared with the negative control (NC) or vaccinated-only group (V). Pathways involved in RNA metabolism (blue text), protein metabolism (green text), citric acid cycle (red text), DNA repair (brown text), mitophagy (black text), and mitochondrial biogenesis (purple text) are shown. Grey solid circles indicate the fold-enrichment in each pathway in the first group compared with the second group in each pair-wise comparison. A false discovery rate (FDR) of < 0.05 was considered significant.

In the CAV-vaccinated-challenged group, there were 12 pathways enriched with genes up-regulated compared with the negative-control group and 21 pathways enriched with genes up-regulated compared with the vaccinated-only group. In the vaccinated-CAV-challenged group, there were 19 pathways enriched with genes up-regulated compared with the negative-control group and 22 pathways enriched with genes up-regulated compared with the vaccinated-only group. In the IBDV-vaccinated-challenged group, 21 and 28 pathways were enriched with genes up-regulated compared with the negative-control and vaccinated-only groups, respectively. In the vaccinated-IBDV-challenged group, 29 pathways were enriched with genes up-regulated compared with the negative-control group and 28 pathways were enriched with genes up-regulated compared with the vaccinated-only group. Of these, 6 pathways were involved in RNA metabolism, 6 in protein metabolism, 5 in the citric acid cycle and electron transport chain, 5 in DNA repair, 3 in mitophagy, and 2 in mitochondrial biogenesis (**Figure 3**).

### Reactome pathways involved in transcription, translation, extracellular and intracellular signalling, and immune responses were enriched with genes down-regulated in the immunosuppressed groups compared with the negative-control and vaccinated-only groups

**Figure 4:**
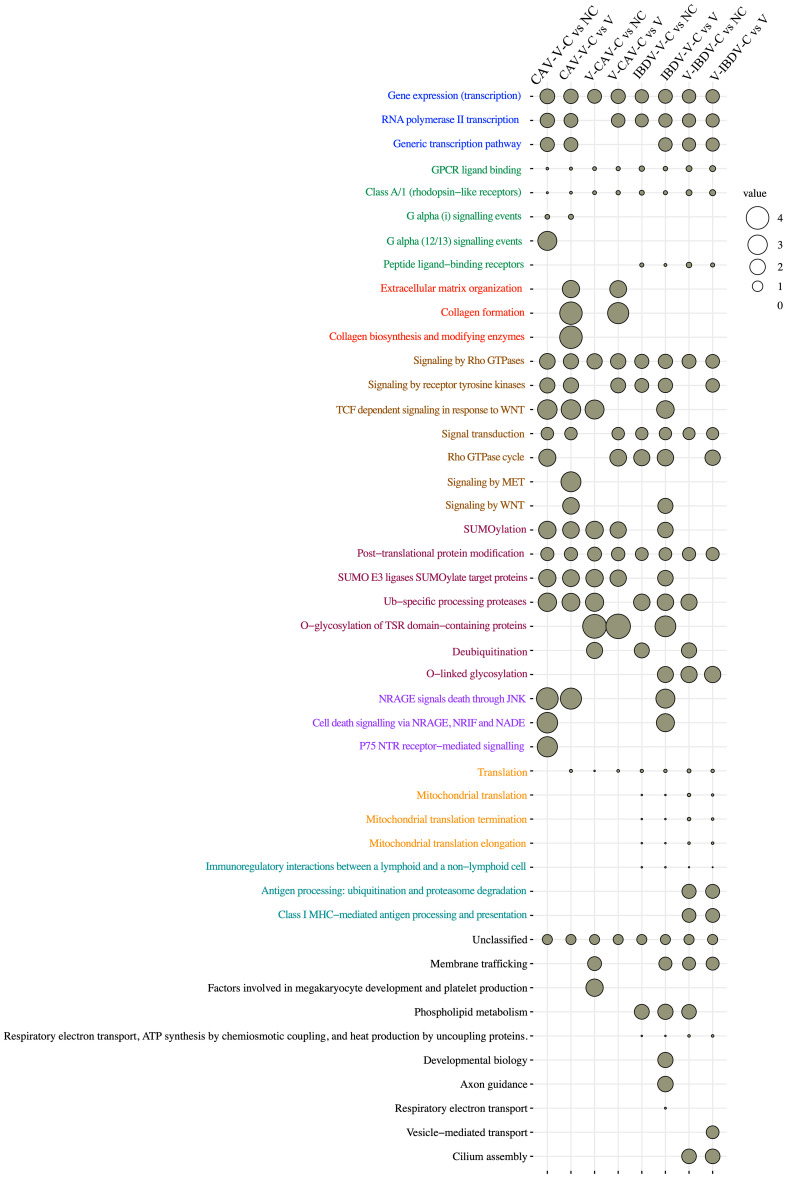
Reactome pathways enriched with down-regulated genes in the four immunosuppressed groups (CAV-V-C; CAV-vaccinated-challenged, V-CAV-C; vaccinated-CAV-challenged, IBDV-V-C; IBDV-vaccinated-challenged, V-IBDV-C; vaccinated-IBDV-challenged) compared with the negative control (N) or vaccinated-only group (V). Pathways involved in transcription (blue text), extracellular signalling (green text), extracellular matrix organisation (red text), intracellular signalling (brown text), post-translational modification of proteins (maroon text), apoptosis (purple text), translation (orange text), immune responses (teal text), and other pathways (black text) are shown. Grey solid circles indicate the fold-enrichment in each pathway in the first group compared with the second group in each pair-wise comparison. A false discovery rate (FDR) of < 0.05 was considered significant.

In the CAV-vaccinated-challenged group, 20 pathways were enriched with genes down-regulated compared with the negative-control group and 22 pathways were enriched with genes down-regulated compared with the vaccinated-only group. In the vaccinated-CAV-challenged group, 17 pathways were enriched with genes down-regulated compared with the negative-control group and 18 pathways were enriched with genes down-regulated compared with the vaccinated-only group. In the IBDV-vaccinated-challenged group, 20 and 32 pathways were enriched with genes down-regulated compared with the negative-control and vaccinated-only groups, respectively. In the vaccinated-IBDV-challenged group, 24 pathways were enriched with genes down-regulated compared with the negative-control group and 24 pathways were enriched with genes down-regulated compared with the vaccinated-only group. Of these, 3 pathways were involved in transcription, 5 in extracellular signalling, 3 in extracellular matrix organisation, 7 in intracellular signalling, 7 in post-translational modifications, 3 in apoptosis, 4 in translation, 3 in immune responses, and 10 in other functions (**Figure 4**). The pathways involved in immune responses and cilium assembly were enriched with genes down-regulated in the two IBDV-infected groups, but not in the two CAV-infected groups (**Figure 4**).

### Reactome pathways involved in DNA replication and cell cycle, immune responses, cell signalling, protein metabolism, haemostasis, and membrane trafficking were enriched with genes down-regulated in the immunosuppressed groups compared with the challenged-only group

**Figure 5:**
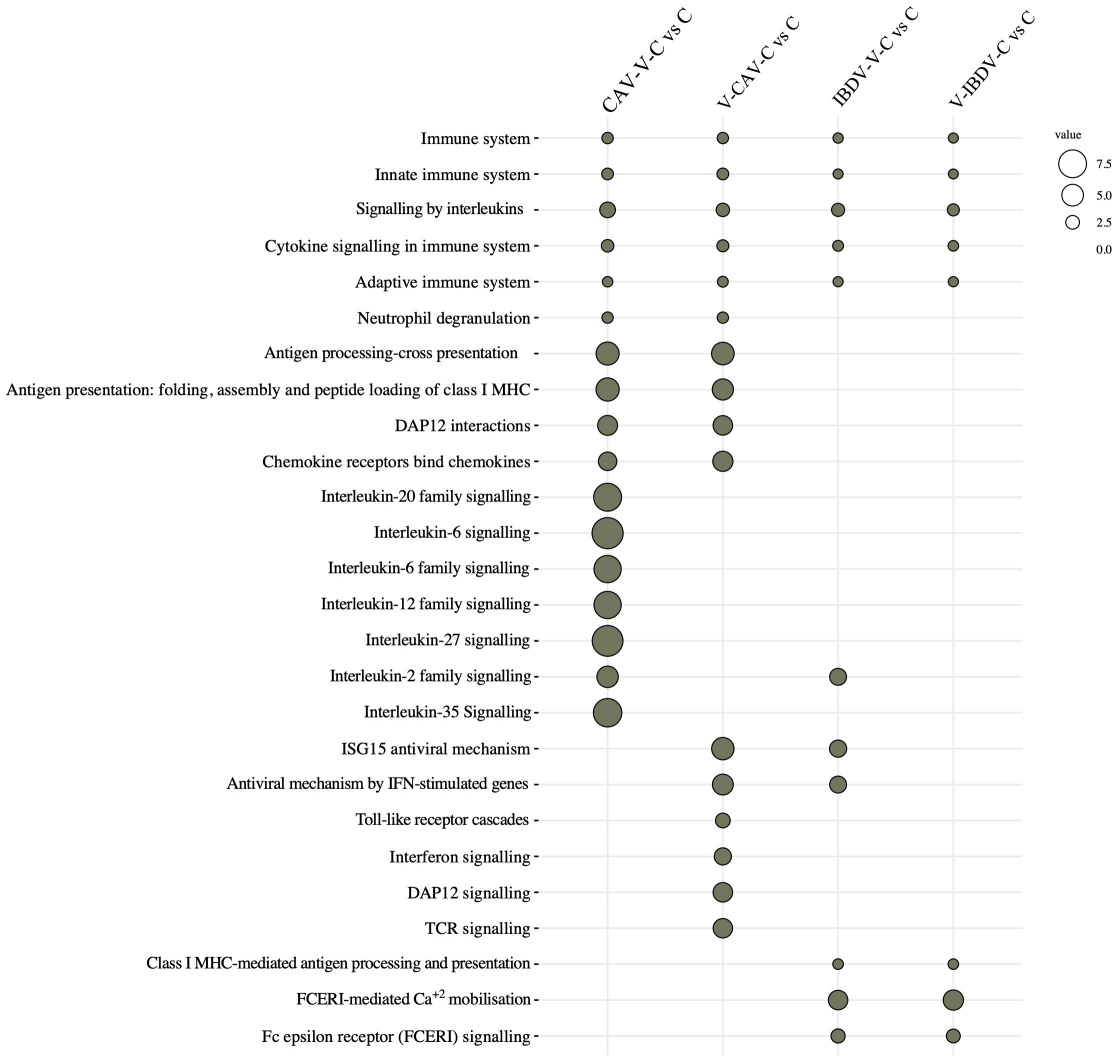
Reactome pathways associated with immune responses that were enriched with down-regulated genes in the four immunosuppressed groups (CAV-V-C; CAV-vaccinated-challenged, V-CAV-C; vaccinated-CAV-challenged, IBDV-V-C; IBDV-vaccinated-challenged, V-IBDV-C; vaccinated-IBDV-challenged) compared with the challenged-only (C) group. Grey solid circles indicate the fold-enrichment in each pathway in the first group compared with the second group in each pair-wise comparison. A false discovery rate (FDR) of < 0.05 was considered significant.

In the CAV-vaccinated-challenged group, 69 pathways were enriched with genes down-regulated compared with the challenged-only group. In the vaccinated-CAV-challenged group, 76 pathways were enriched with genes down-regulated compared with the challenged-only group. In the IBDV-vaccinated-challenged group, 80 pathways were enriched with genes down-regulated compared with the challenged-only group. In the vaccinated-IBDV-challenged group, 67 pathways were enriched with genes down-regulated compared with the challenged-only group. Of these, 38 pathways were involved in DNA replication and cell cycle, 26 in immune responses, 6 in cell signalling, 25 in RNA metabolism, transcription and post-translational modification of proteins, 4 in haemostasis, and 6 in membrane trafficking. Pathways involved in cytokine and chemokine signalling, antigen processing and presentation by class I major histocompatibility complex (MHC) molecules, and receptor signalling were among those related to immune responses that were enriched with down-regulated genes (**Figure 5**). Interleukin (IL) signalling pathways, including the IL-20, IL-6, IL-12, IL-27 and IL-35 signalling pathways, toll-like receptor cascades, and T-cell receptor (TCR) signalling pathways were enriched with genes down-regulated only in CAV-infected groups compared to the challenged-only group (**Figure 5**).

There were no pathways enriched with genes up-regulated in any of the four immunosuppressed groups compared with the challenged-only group.

### Protein classes involved in RNA and protein metabolism and oxidative stress were enriched with up-regulated genes in the immunosuppressed groups compared with negative-control and vaccinated-only groups

**Figure 6:**
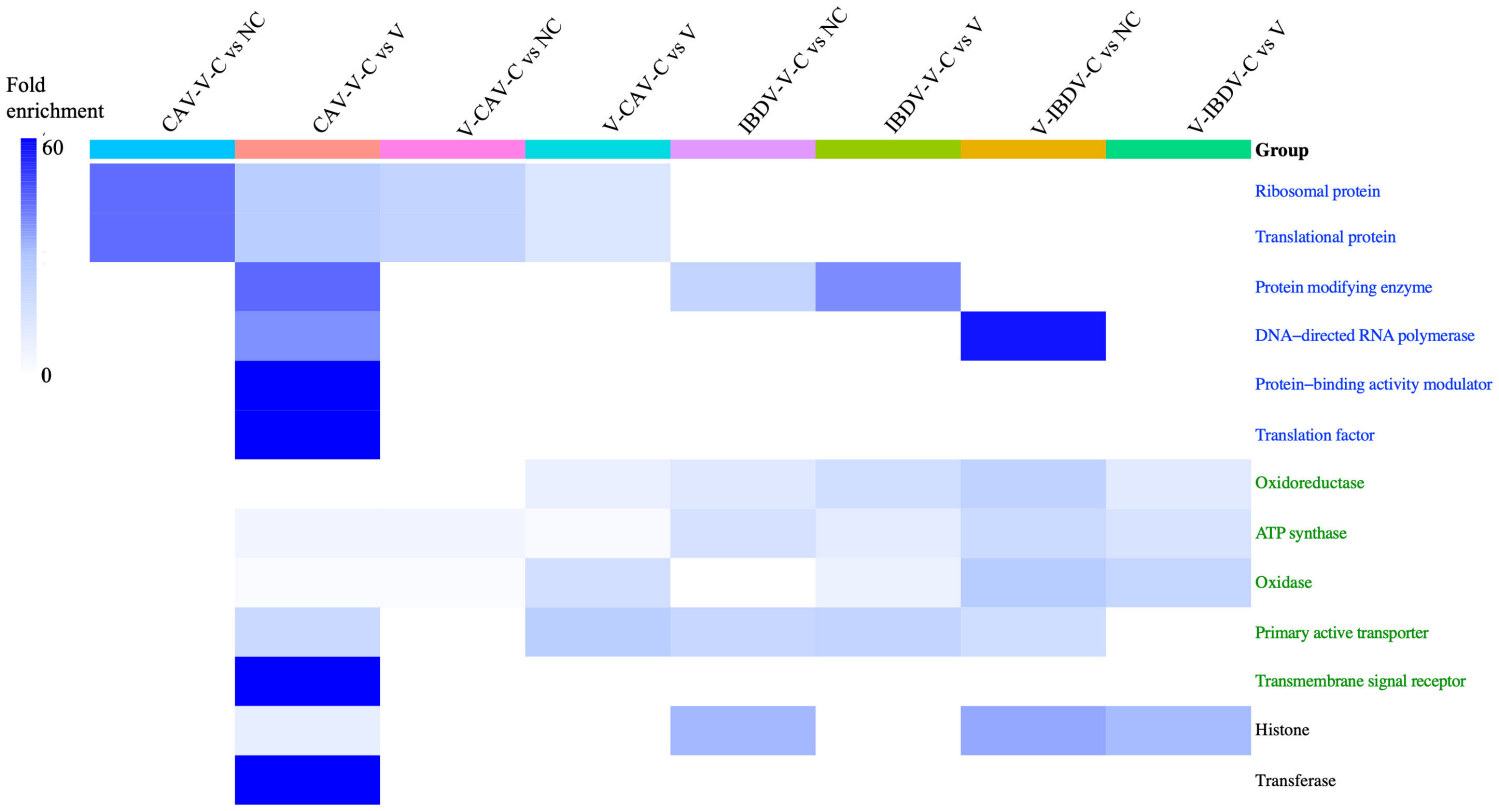
Protein classes enriched with up-regulated genes in the four immunosuppressed groups (CAV-V-C; CAV-vaccinated-challenged, V-CAV-C; vaccinated-CAV-challenged, IBDV-V-C; IBDV-vaccinated-challenged, V-IBDV-C; vaccinated-IBDV-challenged) compared with the negative control (N) or vaccinated-only group (V). Protein classes involved in RNA and protein metabolism (blue text), oxidative stress (green text) and other protein classes (black text) are shown. A false discovery rate (FDR) of < 0.05 was considered significant.

In the CAV-vaccinated-challenged group, two protein classes were enriched with genes up-regulated compared with the negative-control group, and 13 protein classes were enriched with genes up-regulated compared with the vaccinated-only group. In the vaccinated-CAV-challenged group, 6 protein classes were enriched with genes up-regulated compared with the negative-control group and 7 protein classes were enriched with genes up-regulated compared with the vaccinated-only group. In the IBDV-vaccinated-challenged group, 9 protein classes were enriched with genes up-regulated compared with the negative-control group and 8 protein classes were enriched with genes up-regulated compared with the vaccinated-only group. In the vaccinated-IBDV-challenged group, 9 protein classes were enriched with genes up-regulated compared with the negative-control group and 7 protein classes were enriched with genes up-regulated compared with the vaccinated-only group. These protein classes mainly play a role in RNA and protein metabolism, and oxidative stress (**Figure 6**).

### Protein classes involved in immune responses, cell signalling, cell proliferation, organisation of cytoskeleton and extracellular matrix were enriched with genes down-regulated in the immunosuppressed groups compared with negative-control and vaccinated-only groups

**Figure 7:**
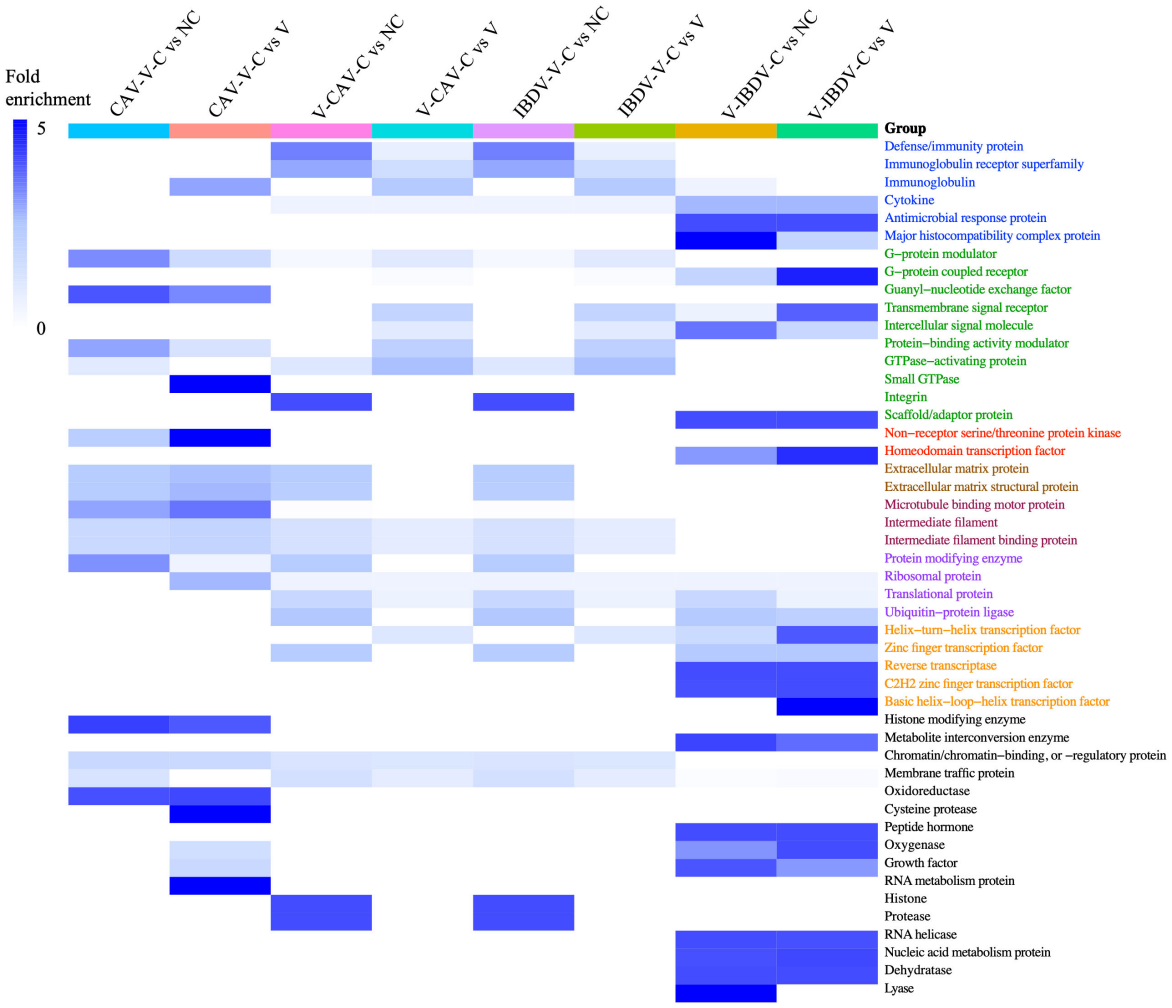
Protein classes enriched with down-regulated genes in the four immunosuppressed groups (CAV-V-C; CAV-vaccinated-challenged, V-CAV-C; vaccinated-CAV-challenged, IBDV-V-C; IBDV-vaccinated-challenged, V-IBDV-C; vaccinated-IBDV-challenged) compared with the negative control (N) or vaccinated-only group (V). Protein classes involved in immune responses (blue text), cell signalling (green text), cell proliferation, differentiation and movement (red text), extracellular matrix (brown text), cytoskeleton (maroon text), translation (purple text), transcription (orange text), and other protein classes (black text) are shown. A false discovery rate (FDR) of < 0.05 was considered significant.

In the CAV-vaccinated-challenged group, 26 protein classes were enriched with genes down-regulated compared with the negative-control group, and 32 protein classes were enriched with genes down-regulated compared with the vaccinated-only group. In the vaccinated-CAV-challenged group, 31 protein classes were enriched with genes down-regulated compared with the negative-control group and 32 protein classes were enriched with genes down-regulated compared with the vaccinated-only group. In the IBDV-vaccinated-challenged group, 36 protein classes were enriched with genes down-regulated compared with the negative-control group and 35 protein classes were enriched with genes down-regulated compared with the vaccinated-only group. In the vaccinated-IBDV-challenged group, 34 protein classes were enriched with genes down-regulated compared with the negative-control group and also the vaccinated-only group. These protein classes mainly play a role in immune responses, cell signalling, cell proliferation, differentiation and movement, extracellular matrix and cellular cytoskeleton organisation, transcription, and translation (**Figure 7**).

### Protein classes involved in nucleic acid metabolism, cell signalling, protein metabolism, organisation of cytoskeleton, and immune responses were enriched with genes down-regulated in the immunosuppressed groups compared with the challenged-only group

**Figure 8:**
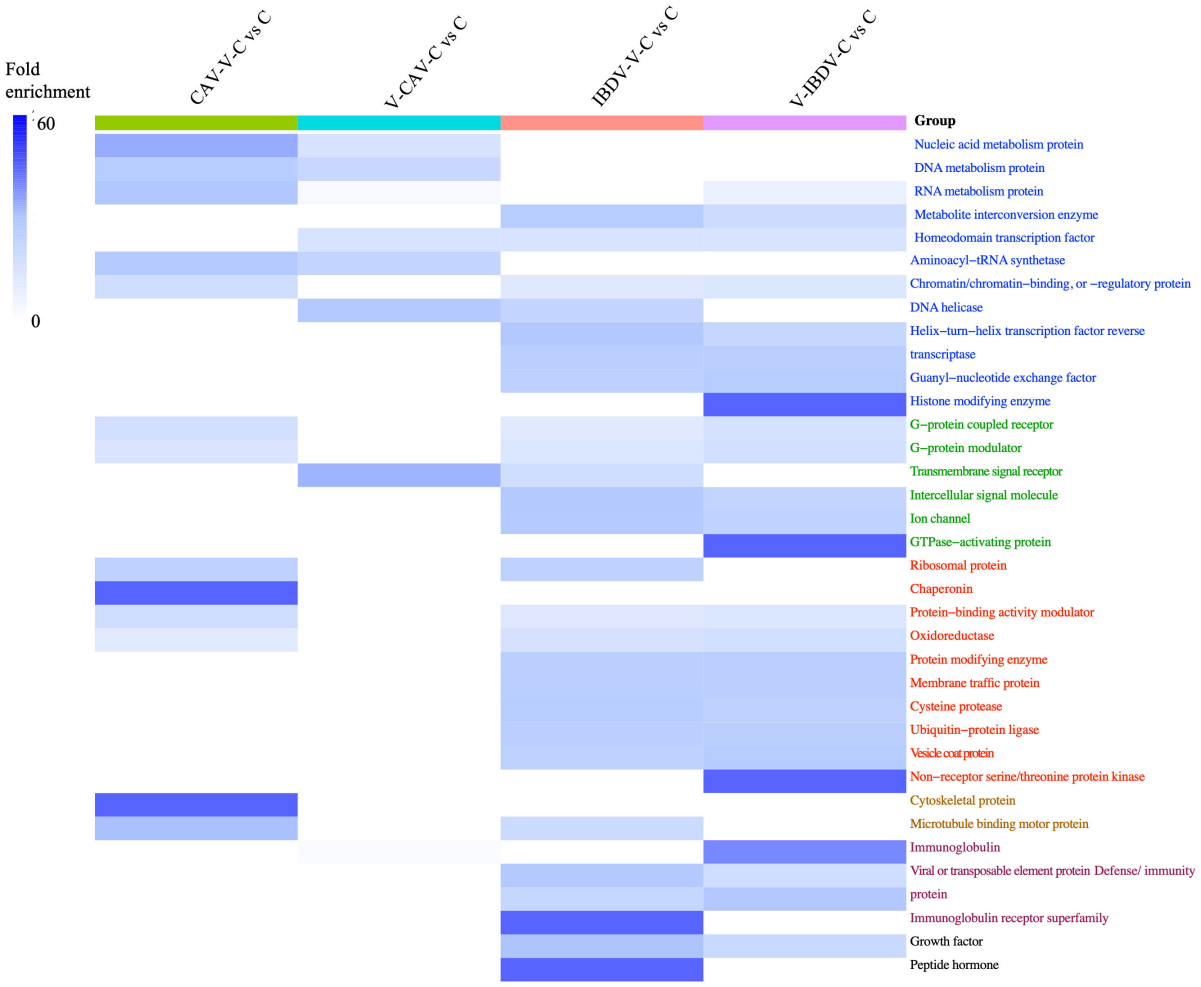
Protein classes enriched with down-regulated genes in the four immunosuppressed groups (CAV-V-C; CAV-vaccinated-challenged, V-CAV-C; vaccinated-CAV-challenged, IBDV-V-C; IBDV-vaccinated-challenged, V-IBDV-C; vaccinated-IBDV-challenged) compared with the challenged-only group (C). Protein classes involved in nucleic acid metabolism (blue text), cell signalling (green text), protein metabolism (red text), cytoskeleton (brown text), immune responses (maroon text), and other protein classes (black text) are shown. A false discovery rate (FDR) of < 0.05 was considered significant.

No protein classes were enriched with genes up-regulated in the immunosuppressed groups compared with the challenged-only group. In the CAV-vaccinated-challenged group, 16 protein classes were enriched with genes down-regulated compared with the challenged-only group. In the vaccinated-CAV-challenged group, 11 protein classes were enriched with genes down-regulated compared with the challenged-only group. In the IBDV-vaccinated-challenged group, 31 protein classes were enriched with genes down-regulated compared with the challenged-only group. In the vaccinated-IBDV-challenged group, 30 protein classes were enriched with genes down-regulated compared with the challenged-only group. These protein classes mainly play a role in nucleic acid metabolism, cell signalling, protein metabolism, cytoskeleton organisation, and immune responses (**Figure 8**).

## Discussion

This study investigated the effects of immunosuppression before or after vaccination with a live attenuated *M. gallisepticum* vaccine on tracheal host responses to subsequent infection with *M. gallisepticum*. The results showed that immunosuppression induced by infection with CAV or IBDV resulted in differences in the transcriptional responses of the tracheal mucosae to infection with *M. gallisepticum* whether the immunosuppression occurred at the time of vaccination or at the time of challenge with wild type *M. gallisepticum*.

Pair-wise comparisons of host gene transcription in the tracheal mucosae of birds in the vaccinated-only, vaccinated-and-challenged and negative-control groups did not identify significant differences between them. In contrast, significant transcriptional differences were identified when the challenged-only group was compared with each of these groups, confirming that the ts-304 vaccine provided protection against transcriptional changes associated with pathological changes in the trachea caused by infection with *M. gallisepticum* (15, 16). The functional attributes associated with the significant changes in transcription in the challenged-only group were generally the same as those detected after chronic infection with *M. gallisepticum* in previous studies (15, 16), so they have not been reiterated here.

The transcriptional data indicated that the immunosuppressed groups were clearly distinct from the immunocompetent groups, including the challenged-only group. Birds in all four immunosuppressed groups had more profound transcriptional changes than the challenged-only birds, even though they had been vaccinated against *M. gallisepticum*. Furthermore, unsupervised clustering of the groups based on their transcriptional profiles placed the four immunosuppressed groups in a distinct cluster, separated from a cluster comprising the negative-control, vaccinated-only and vaccinated-and-challenged groups and another cluster comprising only the challenged-only group. This pattern suggests that immunosuppression with either CAV or IBDV substantially altered the tracheal mucosal response to vaccination and subsequent challenge with *M. gallisepticum*, resulting in altered disease outcomes in immunosuppressed birds (12). The most plausible explanation is that infection with either of these two immunosuppressive viruses interfered with the protection induced by the vaccine. Pathway, GO and protein class enrichment analyses detected down-regulation of innate and adaptive immune responses in the immunosuppressed groups compared with the immunocompetent groups, irrespective of the timing of infection with CAV or IBDV. These finding, together with the lower *M. gallisepticum*-specific serum antibody titres, increased recovery of the wild-type challenge strain and pathological changes in the trachea in these birds reported previously (12), suggested that the generation of both the primary immune response after vaccination, as well the secondary immune response after subsequent infection with wild type *M. gallisepticum*, were adversely affected.

However, it could also reflect the direct impact of the immunosuppression induced by infection with CAV or IBDV on the susceptibility of chickens to the pathological changes induced by infection with *M. gallisepticum*. The majority of the functional attributes of the genes differentially transcribed in the four immunosuppressed groups compared with the negative-control/vaccinated-only groups, as determined by GO, pathway and protein class analyses, were common to all four groups. These shared transcriptional changes are most likely to be a consequence of the effects of immunosuppression on the responses of the birds to challenge with *M. gallisepticum*, rather than a consequence of the direct effects of these viruses in the tracheal mucosa. The shared changes included up-regulation of cellular respiratory cycle, mitochondrial biogenesis, RNA and protein metabolism, oxidative stress and DNA repair and down-regulation of transcription, translation, apoptosis, cell signalling and extracellular matrix and cytoskeleton organisation. Similar changes have been detected previously in chickens with a relatively low tracheal mucosal thickness in the absence of lymphoproliferative changes after infection with *M. gallisepticum* (40). Likewise, in the study described here, immunosuppressed birds had relatively low tracheal mucosal thicknesses compared to the challenged-only birds (12). In the earlier study, these transcriptional profiles were interpreted as representing an early phase of the host response to infection, preceding the dysregulated adaptive response characterised by lymphoproliferation in the trachea (40). In the current study, a similar profile in the immunosuppressed groups is likely to reflect attenuation of immune dysregulation and lymphoproliferative responses by CAV- or IBDV-induced immunosuppression.

Similarly, the GOs, pathways and protein classes enriched with genes differentially transcribed in the four immunosuppressed groups compared with the challenged-only group were similar, but differed from those enriched with genes differentially transcribed compared with the negative-control and vaccinated-only groups. Specifically, there was down-regulation of a greater number of GOs, pathways and protein classes involved in DNA replication and cell cycle, and immune responses, in the comparisons of the four immunosuppressed groups with the challenged-only group than in the comparisons of the four immunosuppressed groups with the negative-control and vaccinated-only groups. The most likely reason for this is the up-regulation of DNA replication and the cell cycle, and immune responses, in the challenged-only group, as has been seen in previous studies (14–16), and down-regulation of them in the four immunosuppressed groups, resulting in greater differences between the immunosuppressed groups and the challenged-only group. Downregulation of immune responses and cellular proliferation, differentiation and migration could also suppress the activity of immune response cells and the immune dysregulation caused by *M. gallisepticum* in the tracheal mucosae of immunosuppressed birds, thereby suppressing the development of the lymphoproliferative lesions that are a major feature of the pathology caused by infection with *M. gallisepticum* in immunocompetent birds. The suppression of lymphoproliferative responses by CAV and IBDV (41–43) is likely to be the reason that the tracheal lesions were less severe in the immunosuppressed birds than in the challenged-only birds, but more severe than in the negative-control, vaccinated-only, and vaccinated-and-challenged birds in our previous study (12). Evidence of downregulation of formation of cilia was only detected in the IBDV-infected groups in comparisons with the negative-control and vaccinated-only groups. In contrast, the GO analysis implied formation of cilia was up-regulated in the immunosuppressed groups in comparisons with the challenged-only group. This is likely to be a reflection of the effects of infection of the tracheal mucosa with *M. gallisepticum* on cilium assembly in the challenged-only group (14–16), and the absence or reduction of these effects in immunosuppressed mucosae.

CAV primarily suppresses cell-mediated immune responses and several key pathways involved in these responses, including DAP12 interactions and signalling, antigen processing and presentation by class I MHC, neutrophil degranulation (heterophils in chickens), toll-like receptor cascades, TCR signalling, interferon signalling, and several cytokine signalling pathways, were down-regulated only in the CAV-infected groups. This suggests that presentation of endogenous antigens to immune effector cells was specifically suppressed in these birds, diminishing activation of cell-mediated immunity. Such suppression is likely to have been primarily driven by the down-regulation of DAP12, which plays a role in triggering toll-like receptors, and other cell-surface receptors on antigen-presenting cells, leading to recognition of invading pathogens and presentation of antigens to effector cells by class I MHC after binding to endogenous ligands (44). Three IL-12 family cytokine signalling pathways (those for IL-12, IL-27 and IL-35) were down-regulated only in the CAV-vaccinated-challenged group. This implied suppression of pathways regulating pro- and anti-inflammatory responses, proliferation of helper T (Th)1 cells and differentiation of regulatory T (Treg) cells, which are mediated by IL-12 and IL-27 (45, 46). In contrast, IL-35 signalling is known to suppress T cell proliferation (47). Furthermore, the IL-6 signalling pathway, which mediates inflammation (48), and IL-20, which is not well characterised in birds, were also downregulated only in the CAV-vaccinated-challenged group.

Down-regulation of toll-like receptor cascades, interferon signalling, DAP12 signalling, and TCR signalling was only seen to the vaccinated-CAV-challenged group. Although there were differences between the two CAV-infected groups in the sets of enriched pathways, these pathways predominantly converged on T cell-mediated immune functions. The differences seen between the groups are likely to be attributable to variations in the timing of CAV infection, as these groups were infected at different time points. Comparable changes in cytokine production, as well as stimulation or suppression of proliferation and differentiation, and receptor signalling of T cells, have been reported previously over the course of CAV infections (19). Collectively, these findings suggest that CAV infection disrupts immune homeostasis in the tracheal mucosa by suppressing both innate (proinflammatory) and downstream adaptive T cell responses. Virus-induced dysregulation of TCR signalling may also represent a potential immune evasion strategy employed by CAV (19).

The effect of infection with IBDV appeared to be more severe than that of infection with CAV, as infection with IBDV resulted in transcriptional changes in a greater number of genes, whether infection with IBDV occurred before or after vaccination. Moreover, only the IBDV-infected groups had significant differences in transcription compared to the vaccinated-and-challenged group. While the number of altered genes alone does not define the biological severity of the response, functional annotation of these changes indicated that, similar to the CAV-infected groups, IBDV infection was associated with down-regulation of T cell-related pathways and antigen processing and presentation via class I MHC. In addition, evidence of down-regulation of ciliary function and cell junction organisation was detected only in the IBDV-infected groups, suggesting that IBDV may further compromise mucosal integrity by interfering with vaccine-induced protection against *M. gallisepticum*-associated pathology. These findings, when considered alongside the more pronounced tracheal lesions and the greater reduction in *M. gallisepticum*-specific serum antibody responses in the IBDV-infected groups compared with the CAV-infected groups described previously (12), support an important role for humoral immunity in conjunction with cell-mediated immunity in protection against virulent *M. gallisepticum* (12, 49–51). Although we did not directly assess B cell depletion or dysfunction in lymphoid organs in these birds, the known tropism of IBDV for B cells provides a plausible explanation for the reduced concentrations of serum antibodies against *M. gallisepticum* in the IBDV-infected chickens (12).

More GOs, pathways and protein classes involved in protein metabolism, including post-translational modification, intracellular transport between the rough endoplasmic reticulum and Golgi complex, vesicle-mediated transport, and membrane trafficking, were downregulated in the two IBDV-infected groups in comparisons with the negative-control, vaccinated-only and challenged-only groups than in comparisons with the CAV-infected groups. However, the specific effects of these changes are uncertain, as it is not clear whether they resulted in reduced expression of particular proteins or effects on a wide range of cellular functions.

This is the first study to investigate genome-wide transcriptional changes in immunosuppressed chickens that have been vaccinated against, and subsequently challenged with, a poultry pathogen. The findings demonstrated altered host responses, including immune responses, in the tracheal mucosae of chickens that had been immunosuppressed by infection with either CAV or IBDV, highlighting the genes and pathways that underlie the reduced efficacy of vaccination against *M. gallisepticum* in immunosuppressed birds.

## Data Availability

RNA-seq data from this study are available in the National Center for Biotechnology Information (NCBI) sequence reads archive (SRA) under the accession number PRJNA694482.
