# Supplementary material for: Effects of immunosuppression on tracheal mucosal responses after infection with Mycoplasma gallisepticum in vaccinated and unvaccinated chickens
Source: Front Immunol. 2026 Jul 23;17:1802515. doi: 10.3389/fimmu.2026.1802515 (PMC13442388; doi:10.3389/fimmu.2026.1802515)
Supplement: Supplementary file 1 [file DataSheet1.docx]

**Figure S1.** Treatments administered to each group of birds in this experiment. CAV, chicken anaemia virus; IBDV, infectious bursal disease virus; ts-304, Vaxsafe MG304 vaccine; and Ap3AS, *M. gallisepticum* wild-type strain Ap3AS. The time points at infection/vaccination/challenge indicate the age of the chickens.

**Table S1**. The number of significantly up- and down-regulated genes in each pairwise comparison.

| **Comparison** | **Up-regulated^†^** | **Down-regulated^†^** |
| --- | --- | --- |
| **Challenged-only vs:** |  |  |
| Negative-control | 477 | 1238 |
| Vaccinated-only | 488 | 1306 |
| Vaccinated-and-challenged | 255 | 446 |
|  |  |  |
| **CAV-vaccinated-challenged vs:** |  |  |
| Negative-control | 112 | 2400 |
| Vaccinated-only | 120 | 2534 |
| Challenged-only | 112 | 1749 |
|  |  |  |
| **Vaccinated-CAV-challenged vs:** |  |  |
| Negative-control | 120 | 2800 |
| Vaccinated-only | 125 | 2824 |
| Challenged-only | 136 | 2031 |
|  |  |  |
| **IBDV-vaccinated-challenged vs:** |  |  |
| Negative-control | 183 | 4617 |
| Vaccinated-only | 198 | 4587 |
| Vaccinated-and-challenged | 0 | 26 |
| Challenged-only | 154 | 3370 |
|  |  |  |
| **Vaccinated-IBDV-challenged vs:** |  |  |
| Negative-control | 176 | 5485 |
| Vaccinated-only | 187 | 5559 |
| Vaccinated-and-challenged | 1 | 242 |
| Challenged-only | 68 | 4042 |

^†^Differentially transcribed genes are reported as up- or down-regulated in the first group (in bold) compared to the second group in each comparison.

CAV, chicken anaemia virus; IBDV, infectious bursal disease virus.

A false discovery rate (FDR) of < 0.05 was considered significant.

**Figure S2**. Groups (differentiated by color) of up-regulated cellular components in the CAV-vaccinated-challenged group compared with the negative-control group based on the summarised GOs. The sizes of the boxes are directly proportional to the significance, with a FDR of < 0.01 considered significant.

**Figure S3**. Groups (differentiated by color) of up-regulated cellular components in the vaccinated-CAV-challenged group compared with the negative-control group based on the summarised GOs. The sizes of the boxes are directly proportional to the significance, with a FDR of < 0.01 considered significant.

**Figure S4**. Groups (differentiated by color) of up-regulated cellular components in the IBDV-vaccinated-challenged group compared with the negative-control group based on the summarised GOs. The sizes of the boxes are directly proportional to the significance, with a FDR of < 0.01 considered significant.

**Figure S5**. Groups (differentiated by color) of up-regulated cellular components in the vaccinated-IBDV-challenged group compared with the negative-control group based on the summarised GOs. The sizes of the boxes are directly proportional to the significance, with a FDR of < 0.01 considered significant.

**Figure S6**. Groups (differentiated by color) of up-regulated molecular functions in the CAV-vaccinated-challenged group compared with the negative-control group based on the summarised GOs. The sizes of the boxes are directly proportional to the significance, with a FDR of < 0.01 considered significant.

**Figure S7**. Groups (differentiated by color) of up-regulated molecular functions in the vaccinated-CAV-challenged group compared with the negative-control group based on the summarised GOs. The sizes of the boxes are directly proportional to the significance, with a FDR of < 0.01 considered significant.

**Figure S8**. Groups (differentiated by color) of up-regulated molecular functions in the IBDV-vaccinated-challenged group compared with the negative-control group based on the summarised GOs. The sizes of the boxes are directly proportional to the significance, with a FDR of < 0.01 considered significant.

**Figure S9**. Groups (differentiated by color) of up-regulated molecular functions in the vaccinated-IBDV-challenged group compared with the negative-control group based on the summarised GOs. The sizes of the boxes are directly proportional to the significance, with a FDR of < 0.01 considered significant.

**Figure S10**. Groups (differentiated by color) of up-regulated biological processes in the CAV-vaccinated-challenged group compared with the negative-control group based on the summarised GOs. The sizes of the boxes are directly proportional to the significance, with a FDR of < 0.01 considered significant.

**Figure S11**. Groups (differentiated by color) of up-regulated biological processes in the vaccinated-CAV-challenged group compared with the negative-control group based on the summarised GOs. The sizes of the boxes are directly proportional to the significance, with a FDR of < 0.01 considered significant.

**Figure S12**. Groups (differentiated by color) of up-regulated biological processes in the IBDV-vaccinated-challenged group compared with the negative-control group based on the summarised GOs. The sizes of the boxes are directly proportional to the significance, with a FDR of < 0.01 considered significant.

**Figure S13**. Groups (differentiated by color) of up-regulated biological processes in the vaccinated-IBDV-challenged group compared with the negative-control group based on the summarised GOs. The sizes of the boxes are directly proportional to the significance, with a FDR of < 0.01 considered significant.

**Figure S14**. Groups (differentiated by color) of down-regulated cellular components in the CAV-vaccinated-challenged group compared with the negative-control group based on the summarised GOs. The sizes of the boxes are directly proportional to the significance, with a FDR of < 0.01 considered significant.

**Figure S15**. Groups (differentiated by color) of down-regulated cellular components in the vaccinated-CAV-challenged group compared with the negative-control group based on the summarised GOs. The sizes of the boxes are directly proportional to the significance, with a FDR of < 0.01 considered significant.

**Figure S16**. Groups (differentiated by color) of down-regulated cellular components in the IBDV-vaccinated-challenged group compared with the negative-control group based on the summarised GOs. The sizes of the boxes are directly proportional to the significance, with a FDR of < 0.01 considered significant.

**Figure S17**. Groups (differentiated by color) of down-regulated cellular components in the vaccinated-IBDV-challenged group compared with the negative-control group based on the summarised GOs. The sizes of the boxes are directly proportional to the significance, with a FDR of < 0.01 considered significant.

**Figure S18**. Groups (differentiated by color) of down-regulated molecular functions in the CAV-vaccinated-challenged group compared with the negative-control group based on the summarised GOs. The sizes of the boxes are directly proportional to the significance, with a FDR of < 0.01 considered significant.

**Figure S19**. Groups (differentiated by color) of down-regulated molecular functions in the vaccinated-CAV-challenged group compared with the negative-control group based on the summarised GOs. The sizes of the boxes are directly proportional to the significance, with a FDR of < 0.01 considered significant.

**Figure S20**. Groups (differentiated by color) of down-regulated molecular functions in the IBDV-vaccinated-challenged group compared with the negative-control group based on the summarised GOs. The sizes of the boxes are directly proportional to the significance, with a FDR of < 0.01 considered significant.

**Figure S21**. Groups (differentiated by color) of down-regulated molecular functions in the vaccinated-IBDV-challenged group compared with the negative-control group based on the summarised GOs. The sizes of the boxes are directly proportional to the significance, with a FDR of < 0.01 considered significant.

**Figure S22**. Groups (differentiated by color) of down-regulated biological processes in the CAV-vaccinated-challenged-only group compared with the negative-control group based on the summarised GOs. The sizes of the boxes are directly proportional to the significance, with a FDR of < 0.01 considered significant.

**Figure S23**. Groups (differentiated by color) of down-regulated biological processes in the vaccinated-CAV-challenged-only group compared with the negative-control group based on the summarised GOs. The sizes of the boxes are directly proportional to the significance, with a FDR of < 0.01 considered significant.

**Figure S24**. Groups (differentiated by color) of down-regulated biological processes in the IBDV-vaccinated-challenged-only group compared with the negative-control group based on the summarised GOs. The sizes of the boxes are directly proportional to the significance, with a FDR of < 0.01 considered significant.

**Figure S25**. Groups (differentiated by color) of down-regulated biological processes in the vaccinated-IBDV-challenged-only group compared with the negative-control group based on the summarised GOs. The sizes of the boxes are directly proportional to the significance, with a FDR of < 0.01 considered significant.

**Figure S26**. Groups (differentiated by color) of up-regulated cellular components in the CAV-vaccinated-challenged group compared with the challenged-only group based on the summarised GOs. The sizes of the boxes are directly proportional to the significance, with a FDR of < 0.01 considered significant.

**Figure S27**. Groups (differentiated by color) of up-regulated cellular components in the vaccinated-CAV-challenged group compared with the challenged-only group based on the summarised GOs. The sizes of the boxes are directly proportional to the significance, with a FDR of < 0.01 considered significant.

**Figure S28**. Groups (differentiated by color) of up-regulated cellular components in the IBDV-vaccinated-challenged group compared with the challenged-only group based on the summarised GOs. The sizes of the boxes are directly proportional to the significance, with a FDR of < 0.01 considered significant.

**Figure S29**. Groups (differentiated by color) of up-regulated cellular components in the vaccinated-IBDV-challenged group compared with the challenged-only group based on the summarised GOs. The sizes of the boxes are directly proportional to the significance, with a FDR of < 0.01 considered significant.

**Figure S30**. Groups (differentiated by color) of up-regulated molecular functions in the CAV-vaccinated-challenged group compared with the challenged-only group based on the summarised GOs. The sizes of the boxes are directly proportional to the significance, with a FDR of < 0.01 considered significant.

**Figure S31**. Groups (differentiated by color) of up-regulated molecular functions in the IBDV-vaccinated-challenged group compared with the challenged-only group based on the summarised GOs. The sizes of the boxes are directly proportional to the significance, with a FDR of < 0.01 considered significant.

**Figure S32**. Groups (differentiated by color) of up-regulated molecular functions in the vaccinated-IBDV-challenged group compared with the challenged-only group based on the summarised GOs. The sizes of the boxes are directly proportional to the significance, with a FDR of < 0.01 considered significant.

**Figure S33**. Groups (differentiated by color) of up-regulated biological processes in the CAV-vaccinated-challenged group compared with the challenged-only group based on the summarised GOs. The sizes of the boxes are directly proportional to the significance, with a FDR of < 0.01 considered significant.

**Figure S34**. Groups (differentiated by color) of up-regulated biological processes in the vaccinated-CAV-challenged group compared with the challenged-only group based on the summarised GOs. The sizes of the boxes are directly proportional to the significance, with a FDR of < 0.01 considered significant.

**Figure S35**. Groups (differentiated by color) of up-regulated biological processes in the IBDV-vaccinated-challenged group compared with the challenged-only group based on the summarised GOs. The sizes of the boxes are directly proportional to the significance, with a FDR of < 0.01 considered significant.

**Figure S36**. Groups (differentiated by color) of up-regulated biological processes in the vaccinated-IBDV-challenged group compared with the challenged-only group based on the summarised GOs. The sizes of the boxes are directly proportional to the significance, with a FDR of < 0.01 considered significant.

**Figure S37**. Groups (differentiated by color) of down-regulated cellular components in the CAV-vaccinated-challenged group compared with the challenged-only group based on the summarised GOs. The sizes of the boxes are directly proportional to the significance, with a FDR of < 0.01 considered significant.

**Figure S38**. Groups (differentiated by color) of down-regulated cellular components in the vaccinated-CAV-challenged group compared with the challenged-only group based on the summarised GOs. The sizes of the boxes are directly proportional to the significance, with a FDR of < 0.01 considered significant.

**Figure S39**. Groups (differentiated by color) of down-regulated cellular components in the IBDV-vaccinated-challenged group compared with the challenged-only group based on the summarised GOs. The sizes of the boxes are directly proportional to the significance, with a FDR of < 0.01 considered significant.

**Figure S40**. Groups (differentiated by color) of down-regulated cellular components in the vaccinated-IBDV-challenged group compared with the challenged-only group based on the summarised GOs. The sizes of the boxes are directly proportional to the significance, with a FDR of < 0.01 considered significant.

**Figure S41**. Groups (differentiated by color) of down-regulated molecular functions in the CAV-vaccinated-challenged group compared with the challenged-only group based on the summarised GOs. The sizes of the boxes are directly proportional to the significance, with a FDR of < 0.01 considered significant.

**Figure S42**. Groups (differentiated by color) of down-regulated molecular functions in the vaccinated-CAV-challenged group compared with the challenged-only group based on the summarised GOs. The sizes of the boxes are directly proportional to the significance, with a FDR of < 0.01 considered significant.

**Figure S43**. Groups (differentiated by color) of down-regulated molecular functions in the IBDV-vaccinated-challenged group compared with the challenged-only group based on the summarised GOs. The sizes of the boxes are directly proportional to the significance, with a FDR of < 0.01 considered significant.

**Figure S44**. Groups (differentiated by color) of down-regulated molecular functions in the vaccinated-IBDV-challenged group compared with the challenged-only group based on the summarised GOs. The sizes of the boxes are directly proportional to the significance, with a FDR of < 0.01 considered significant

**Figure S45**. Groups (differentiated by color) of down-regulated biological processes in the CAV-vaccinated-challenged group compared with the challenged-only group based on the summarised GOs. The sizes of the boxes are directly proportional to the significance, with a FDR of < 0.01 considered significant.

**Figure S46**. Groups (differentiated by color) of down-regulated biological processes in the vaccinated-CAV-challenged group compared with the challenged-only group based on the summarised GOs. The sizes of the boxes are directly proportional to the significance, with a FDR of < 0.01 considered significant.

**Figure S47**. Groups (differentiated by color) of down-regulated biological processes in the IBDV-vaccinated-challenged group compared with the challenged-only group based on the summarised GOs. The sizes of the boxes are directly proportional to the significance, with a FDR of < 0.01 considered significant.

**Figure S48**. Groups (differentiated by color) of down-regulated biological processes in the vaccinated-IBDV-challenged group compared with the challenged-only group based on the summarised GOs. The sizes of the boxes are directly proportional to the significance, with a FDR of < 0.01 considered significant.
